# Preoperative prediction of the Lauren classification in gastric cancer using automated nnU-Net and radiomics: a multicenter study

**DOI:** 10.1186/s13244-025-01923-9

**Published:** 2025-02-25

**Authors:** Bo Cao, Jun Hu, Haige Li, Xuebing Liu, Chang Rong, Shuai Li, Xue He, Xiaomin Zheng, Kaicai Liu, Chuanbin Wang, Wei Guo, Xingwang Wu

**Affiliations:** 1 Department of Radiology, The First Affiliated Hospital of Anhui Medical University, 230022 Hefei, People’s Republic of China; 2 Department of Radiology, The Second Affiliated Hospital of Nanjing Medical University, 210011 Nanjing, People’s Republic of China; 3 Department of Radiology, Anhui Provincial Children’s Hospital, Children’s Hospital of Fudan University Anhui Hospital, 230051 Hefei, People’s Republic of China; 4 Department of Pathology, The Second Affiliated Hospital of Nanjing Medical University, 210011 Nanjing, People’s Republic of China; 5 Department of Radiology, The First Affiliated Hospital of USTC, Division of Life Sciences and Medicine, University of Science and Technology of China, 230031 Hefei, People’s Republic of China

**Keywords:** Gastric cancer, Deep learning, Radiomics, Computed tomography

## Abstract

**Objectives:**

To develop and validate a deep learning model based on nnU-Net combined with radiomics to achieve autosegmentation of gastric cancer (GC) and preoperative prediction via the Lauren classification.

**Methods:**

Patients with a pathological diagnosis of GC were retrospectively enrolled in three medical centers. The nnU-Net autosegmentation model was developed using manually segmented datasets and evaluated by the Dice similarity coefficient (DSC). The CT images were processed by the nnU-Net model to obtain autosegmentation results and extract radiomic features. The least absolute shrinkage and selection operator (LASSO) method selects optimal features for calculating the Radscore and constructing a radiomic model. Clinical characteristics and the Radscore were integrated to construct a combined model. Model performance was evaluated via the receiver operating characteristic (ROC) curve.

**Results:**

A total of 433 GC patients were divided into the training set, internal validation set, external test set-1, and external test set-2. The nnU-Net model achieved a DSC of 0.79 in the test set. The areas under the curve (AUCs) of the internal validation set, external test set-1, and external test set-2 were 0.84, 0.83, and 0.81, respectively, for the radiomic model; and 0.81, 0.81, and 0.82, respectively, for the combined model. The AUCs of the radiomic and combined models showed no statistically significant difference (*p* > 0.05). The radiomic model was selected as the optimal model.

**Conclusions:**

The nnU-Net model can efficiently and accurately achieve automatic segmentation of GCs. The radiomic model can preoperatively predict the Lauren classification of GC with high accuracy.

**Critical relevance statement:**

This study highlights the potential of nnU-Net combined with radiomics to noninvasively predict the Lauren classification in gastric cancer patients, enhancing personalized treatment strategies and improving patient management.

**Key Points:**

- The Lauren classification influences gastric cancer treatment and prognosis.
- The nnU-Net model reduces doctors’ manual segmentation errors and workload.
- Radiomics models aid in preoperative Lauren classification prediction for patients with gastric cancer.

**Graphical Abstract:**

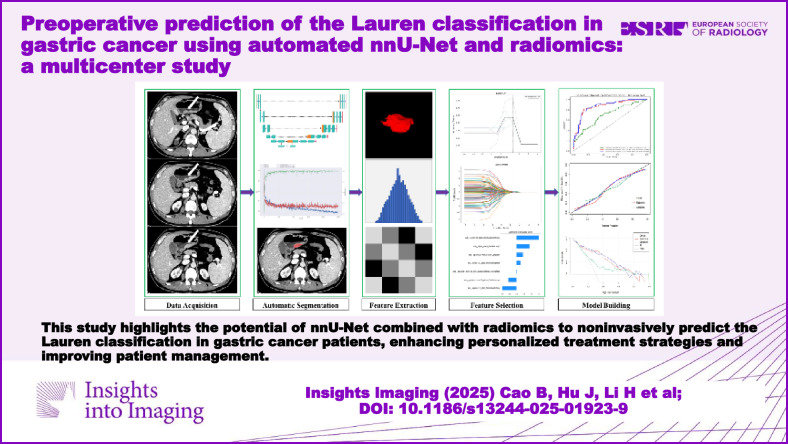

## Introduction

Gastric cancer (GC) is the fifth most common cancer and the fourth leading cause of cancer death worldwide [1]. The Lauren classification system categorizes GC into two main types, namely, intestinal and diffuse types, which differ significantly in terms of pathological characteristics, prognosis, and treatment response [2–5]. The Lauren classification provides clinical value for treatment decisions, with intestinal-type GC typically responding better to chemotherapy and having a better prognosis than diffuse-type GC does. Therefore, accurate preoperative prediction of the Lauren classification is crucial for formulating personalized treatment strategies.

Currently, the main methods for preoperatively predicting the Lauren classification of GC include endoscopy, pathological biopsy, and molecular biomarker analysis. Endoscopy and biopsy provide accurate histological information and are crucial for diagnosis and treatment [6]. However, these techniques have limitations, such as invasiveness, sampling errors, and contraindications in some patients. Noninvasive approaches, such as imaging techniques, are being explored as alternatives for preoperative Lauren classification prediction. However, these methods are still under investigation, and their clinical utility requires further validation [7, 8].

Radiomics extracts many quantitative features from medical images, reflecting tumor heterogeneity and biological behavior [9, 10]. In recent years, radiomics has shown great potential in tumor diagnosis, prognosis prediction, and treatment response assessment [11–15]. Traditional radiomic methods, which rely on the manual delineation of regions of interest (ROIs), are subjective and time-consuming [16]. Automated segmentation techniques have emerged to address this issue. nnU-Net, a neural network based on the U-Net architecture, is specifically designed for medical image segmentation and can automatically adapt to different datasets, achieving efficient and accurate segmentation [17]. Although nnU-Net has performed well in various medical image segmentation tasks, its application in predicting Lauren classification in GC remains to be explored.

This study focuses on the use of an automated nnU-Net model to segment GCs from preoperative CT images and employs radiomic techniques to predict the Lauren classification, specifically distinguishing between the intestinal and diffuse types. The mixed-type Lauren classification is excluded because of its greater aggressiveness, increased lymph node metastasis, and different biological behavior than the intestinal and diffuse types, which could introduce complexities in model training and prediction [18, 19]. By incorporating multicenter data for external validation, we aimed to assess the generalizability and clinical applicability of the model. This approach aims to enhance the automation of segmentation and feature extraction, reduce subjective biases and workload, and provide a reliable and accurate model for the preoperative evaluation of GC.

## Materials and methods

This retrospective multicenter study was approved by the Institutional Review Boards (IRBs) of Center 1, Center 2, and Center 3. Informed consent was waived because of the retrospective nature of this study.

### Study sample and design

**Fig. 1:**
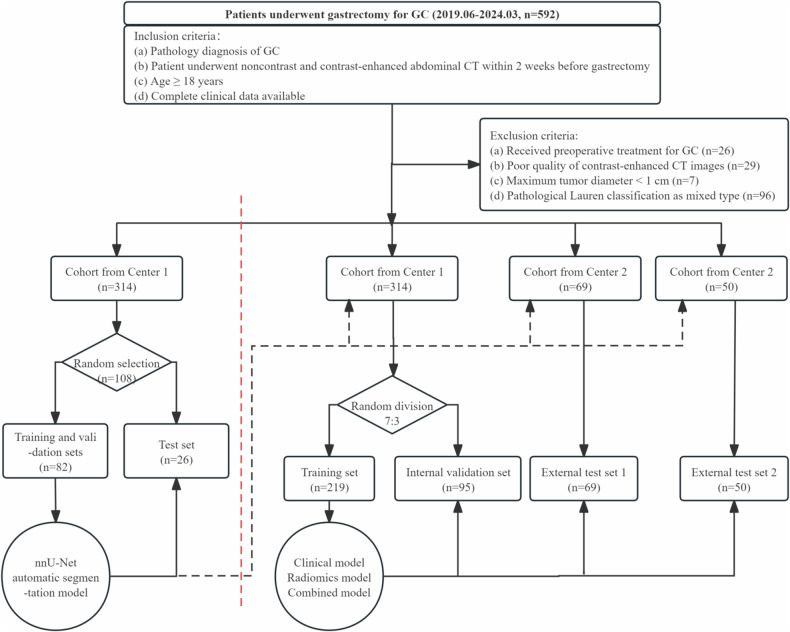
The flowchart of patient selection and study design

From June 2019 to March 2024, a total of 592 patients from three medical centers were screened. Based on the inclusion and exclusion criteria, 433 patients were ultimately included. The inclusion criteria were as follows: (1) patients with pathologically confirmed GC; (2) patients who underwent standard abdominal plain and enhanced CT scans within two weeks before surgery; (3) patients aged ≥ 18 years; and (4) patients whose complete clinical data were available. The exclusion criteria were as follows: (1) received any antitumor treatment before surgery; (2) poor quality of enhanced CT images, such as unfilled stomach or significant motion artifacts; (3) maximum tumor diameter < 1 cm, as lesions smaller than 1 cm are challenging to delineate precisely and can lead to reduced reliability of radiomic feature extraction, which is crucial for building accurate predictive models [11, 20]; and (4) pathological Lauren classification as a mixed type. From the Center 1 cohort (*n* = 314), 108 patients were randomly selected to train the nnU-Net model for automatic tumor segmentation. The Center 1 cohort was also used to train the clinical, radiomic, and combined clinical-radiomic models for the preoperative prediction of the Lauren classification in patients with GC, which were divided into a training set (*n* = 219) and an internal validation set (*n* = 95) at a 7:3 ratio. The cohorts from Center 2 (*n* = 69) and Center 3 (*n* = 50) were used for external testing of the models. The patient selection and study design workflow are illustrated in Fig. 1.

### CT imaging study protocol

All patients fasted for at least 6 hours before the CT examination and drank approximately 1000 mL of water to distend the stomach. CT examinations were performed using either 64- or 128-slice scanners from different manufacturers, including Revolution CT, Optima CT680, and Discovery CT750 from GE Healthcare; IQon Spectral from Philips Health care; and Somatom Definition Flash from Siemens Healthineers. The CT scan parameters were as follows: tube voltages of 100–120 kV, tube currents of 120–640 mA, detector collimations of 0.6–0.625 mm, image matrix of 512 × 512, and slice thicknesses of 1–1.25 mm. Patients were scanned in the supine position for both plain and contrast-enhanced scans, covering the upper abdomen or whole abdomen. To standardize variations in CT slice thickness, all original images were reconstructed to a slice thickness of 5 mm before analysis to ensure data consistency. A nonionic contrast agent (Ultravist 300 or Ioversol 320) was injected at a rate of 3.0 mL/s at doses ranging from 1–1.5 mL/kg. Images were acquired at 20–30 s (arterial phase, AP), 60–70 s (portal venous phase, PP), and 180 s (delayed phase, DP) after contrast injection.

### Assessment of clinical and radiological characteristics

Through the electronic health records (EHR) system, the clinical and pathological data of patients, including sex, age, smoking history, alcohol consumption history, hypertension status, diabetes status, carcinoembryonic antigen (CEA), cancer antigen 125 (CA125), carbohydrate antigen 19-9 (CA19-9), and Lauren classification were collected. Abdominal CT images were obtained through the Picture Archiving and Communication System (PACS). Two radiologists (with 10 and 20 years of experience) independently and blindly assessed the radiological characteristics of all the images. Discrepancies were resolved through consensus discussions. The radiological characteristic measured was the maximum tumor diameter, defined as the longest linear distance measured on the largest cross-sectional area of the tumor on axial images [21].

### Manual lesion segmentation

The CT images of the portal venous phase of 108 randomly selected patients from the Center 1 cohort were manually segmented using ITK-SNAP software (version 3.8.0; http://www.itksnap.org) [22] by two radiologists. The two radiologists delineated the lesions together, avoiding the inclusion of blood vessels and gastric contents. All annotations were reviewed by a third radiologist with 20 years of experience.

### Construction of the automatic segmentation model

**Fig. 2:**
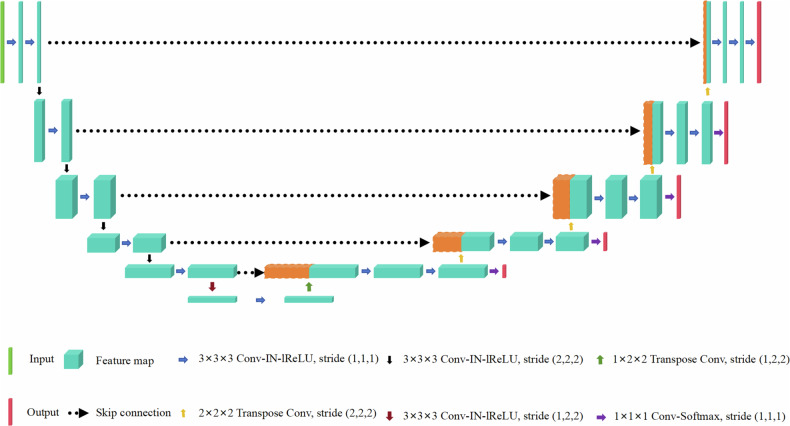
Network architecture of nnU-Net for automatic segmentation of GC lesions

The nnU-Net is a deep learning model designed for efficient segmentation by automatically configuring the segmentation pipeline. The code used in this study is available at http://github.com/MIC-DKFZ/nnUNet. We utilized the default configuration of nnU-Net and trained our model on the 3D full-resolution U-Net architecture. The network architecture of nnU-Net is depicted in Fig. 2.

This study included a total of 108 patients with CT images and their corresponding manual segmentation labels, which were divided into training and validation sets (*n* = 82) and a test set (*n* = 26). Fivefold cross-validation was employed to fine-tune the model during the training process, and model performance was evaluated on the test set. The evaluation metrics included the dice similarity coefficient (DSC), intersection over union (IoU), precision, and recall.

During the data preprocessing stage, the nnU-Net framework standardizes the original images on the basis of task characteristics. First, the images were resampled to a voxel size of 5.0 × 0.78 × 0.78 mm^3^ to optimize segmentation performance. Next, the image intensities were clipped to the range of 0.5% to 99.5% to reduce outliers. Finally, Z score normalization was applied to standardize the intensities to zero mean and unit variance, ensuring compatibility with the model. To enhance the model’s generalization ability, various data augmentation strategies, including rotation, scaling, and random cropping, were applied during training. The model was trained using a combination of Dice loss and cross-entropy loss functions, with a batch size of 2. The initial learning rate was set to 0.01, and the Adam optimizer was used, with a stochastic gradient descent strategy and a Nesterov momentum of 0.99. The model was trained for a total of 1000 epochs without employing an early stopping strategy. The entire training and inference process was implemented using the PyTorch framework on a Windows operating system with an NVIDIA 4090 RTX GPU.

### Radiomics feature extraction

**Fig. 3:**
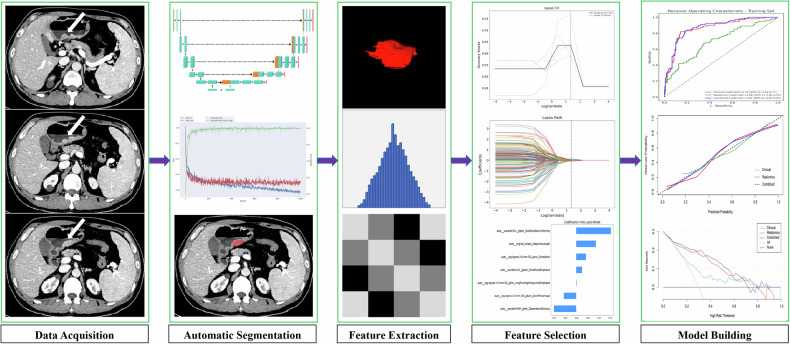
The radiomics diagram of the study

Figure 3 illustrates the workflow of the radiomic study. The nnU-Net model was used to automatically segment the CT images of the portal venous phase, obtaining a three-dimensional region of interest (3D-ROI) for each patient. To ensure consistency, the segmented images and corresponding original CT images were resampled to a fixed voxel size of 1.0 × 1.0 × 1.0 mm³ using SimpleITK (version 2.1.1; https://simpleitk.org). The gray-level intensity values of the resampled images were normalized using *Z* score normalization [23] to minimize variability across datasets.

Radiomic features were extracted from each 3D-ROI using PyRadiomics software [24] (version 3.0.1; https://pypi.org/project/pyradiomics/). The feature extraction process adhered to the guidelines of the Imaging Biomarker Standardization Initiative (IBSI) [25]. A total of 1874 radiomic features were extracted for each patient, including first-order features (360), a gray-level cooccurrence matrix (GLCM, 480), a gray-level dependence matrix (GLDM, 280), a gray-level run length matrix (GLRLM, 320), a gray-level size zone matrix (GLSZM, 320), a neighboring gray-tone difference matrix (NGTDM, 100), and shape-based features (14).

### Radiomics feature selection and model building

Feature selection was performed on the training set. First, collinear features with Pearson correlation coefficients (absolute values) ≥ 0.9 were eliminated. Then, features with *p* < 0.05 were retained using SelectKBest. Finally, the least absolute shrinkage and selection operator (LASSO) regression algorithm was used to select the optimal features by adjusting the penalty parameter *λ*, and the radiomics score (Radscore) was calculated based on the LASSO coefficients. A logistic regression (LR) classifier was used to construct the radiomic model. Univariate and multivariate LR analyses were conducted on the clinical and radiological features to identify independent predictors of the Lauren classification and construct a clinical model. The combined clinical-radiomics model was built by integrating the independent predictors and the Radscore.

### Statistical analysis

Normally distributed continuous variables are expressed as the means ± standard deviations and were compared using the independent samples *t*-test. Nonnormally distributed data are presented as medians (interquartile ranges, M [P25, P75]) and were compared using the Mann‒Whitney *U*-test for two groups or the Kruskal‒Wallis *H*-test for multiple groups. Categorical variables are expressed as frequencies (percentages) and were compared using the *χ*² test or Fisher’s exact test. Univariate and multivariate LR analyses were conducted to identify independent predictors. The accuracy of the automatic segmentation model was quantified using the DSC. Model performance was evaluated by plotting receiver operating characteristic (ROC) curves and calculating the area under the curve (AUC), calibration curves, and decision curve analysis (DCA). The DeLong test was used to compare the performance of different models. Statistical analyses were performed using SPSS (version 27.0) and R software (version 3.5.3). A two-sided *p* < 0.05 was considered statistically significant.

## Results

### Baseline characteristics of patients

Among the 592 patients who underwent gastrectomy for GC, 26 were excluded because of preoperative antitumor treatment, 29 because of poor image quality, 7 because of a maximum tumor diameter < 1 cm, and 96 because of mixed-type Lauren classification (Fig. 1). A total of 433 GC patients with an age range of 26-90 years (median age 69 years (IQR, 59–72.5 years)), of whom 322 were male (74.4%) and 111 were female (25.6%), were included in this study. The histological results revealed that the Lauren classification included 228 cases of the intestinal type (52.7%) and 205 cases of the diffuse type (47.3%). The distribution of the Lauren classification was balanced across the training, internal validation, and external test sets 1 and 2 (*p* = 0.649), indicating that there was no significant difference in classification distribution between the different datasets. This enhances the reliability of model training and validation, ensuring the generalizability and robustness of the study results. All baseline characteristics are detailed in Table 1.

**Table 1:** Baseline characteristics of patients

Characteristics	Training set (*n* = 219)	Internal validation set (*n* = 95)	External test set-1 (*n* = 69)	External test set-2 (*n* = 50)	*p-*value
Age (years), median (IQR)	68.0 (60.0, 74.0)	65.0 (58.0, 72.0)	64.0 (53.5, 69.0)	70.0 (60.8, 72.3)	< 0.001
Gender, no. (%)					0.971
Female	55 (25.1)	25 (26.3)	17 (24.6)	14 (28.0)	
Male	164 (74.9)	70 (73.7)	52 (75.4)	36 (72.0)	
Smoking history, no. (%)					0.724
Nonsmoker	166 (75.8)	67 (70.5)	52 (75.4)	39 (78.0)	
Smoker	53 (24.2)	28 (29.5)	17 (24.6)	11 (22.0)	
Drinking history, no. (%)					0.904
Non-drinker	172 (78.5)	73 (76.8)	55 (79.7)	41 (82.0)	
Drinker	47 (21.5)	22 (23.2)	14 (20.3)	9 (18.0)	
Hypertension, no. (%)					0.315
Yes	72 (32.9)	27 (28.4)	19 (27.5)	10 (20.0)	
No	147 (67.1)	68 (71.6)	50 (72.5)	40 (80.0)	
Diabetes, no. (%)					0.256
Yes	27 (12.3)	8 (8.4)	3 (4.3)	5 (10.0)	
No	192 (87.7)	87 (91.6)	66 (95.7)	45 (90.0)	
CEA (ng/mL), median (IQR)	2.52 (1.46, 4.88)	2.24 (1.21, 4.41)	2.52 (1.59, 4.01)	2.57 (1.26, 3.86)	0.612
CA125 (U/mL), median (IQR)	9.83 (6.59, 14.82)	9.25 (6.84, 14.21)	7.53 (5.49, 11.09)	8.95 (6.17, 11.48)	0.024
CA19-9 (U/mL), median (IQR)	11.59 (5.97, 30.16)	10.57 (6.63, 28.44)	8.60 (4.35, 16.08)	10.60 (6.11, 18.05)	0.152
Maximum tumor diameter (cm), median (IQR)	4.80 (3.50, 6.50)	5.20 (4.00, 6.50)	5.00 (3.50, 7.50)	5.75 (4.45, 8.00)	0.031
Lauren type, no. (%)					0.649
Intestinal type	119 (54.3)	52 (54.7)	32 (46.4)	25 (50.0)	
Diffuse type	100 (45.7)	43 (45.3)	37 (53.6)	25 (50.0)	

*IQR* interquartile range, *CEA* carcinoma embryonic antigen, *CA125* cancer antigen 125, *CA19-9* cancer antigen 19-9

### Performance evaluation of the nnU-Net automatic segmentation model

The performance of the automatic segmentation model was evaluated through 5-fold cross-validation in the training and validation sets. The best segmentation results for the DSC, IoU, precision, and recall metrics were 80.99%, 68.22%, 85.77%, and 76.80%, respectively. For the test set, the DSC, IoU, precision, and recall values were 79.79%, 66.59%, 84.91%, and 73.89%, respectively (Table 2). Supplementary Fig. 1 shows comparison examples of manual and automatic segmentation of lesions in GC patients.

**Table 2:** Fivefold cross-validation and test set results of the automatic segmentation model

Performance metric	Fold 0	Fold 1	Fold 2	Fold 3	Fold 4	Test Set
DSC (%)	77.19	75.27	80.99	80.14	75.80	79.79
IoU (%)	63.11	61.94	68.22	67.06	61.38	66.59
Precision (%)	79.48	84.54	85.77	85.88	77.85	84.91
Recall (%)	74.45	66.40	76.80	73.26	70.49	73.89

*DSC* dice similarity coefficient, *IoU* intersection over union

### Results of clinical feature selection and model evaluation

**Fig. 4:**
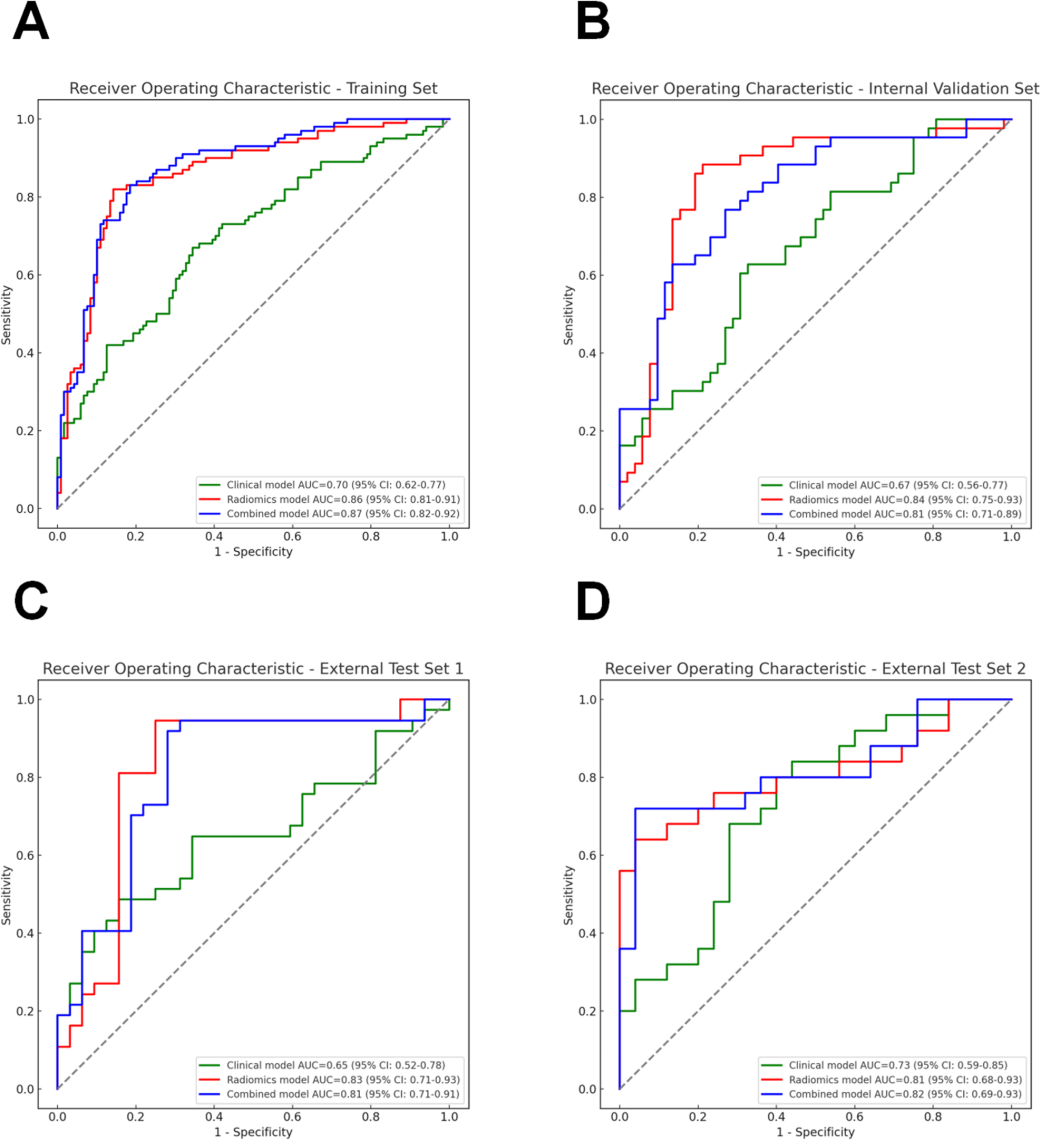
Comparison of ROC curves for different models. **A** Training set; **B** Internal validation set; **C** External test set-1; **D** External test set-2. The legend shows the AUC values and 95% confidence intervals (CI). The green, red, and blue lines represent the clinical model, radiomics model, and combined model, respectively

Univariate analysis of the training set revealed that age, CA125 level, and maximum tumor diameter were associated with the Lauren classification (*p* < 0.05). Multivariate logistic regression analysis further indicated that age (OR = 0.95; 95% CI [0.92, 0.98]; *p* = 0.003), CA125 level (OR = 1.05; 95% CI [1.01, 1.10]; *p* = 0.015), and maximum tumor diameter (OR = 1.27; 95% CI [1.11, 1.46]; *p* < 0.001) were independent predictors of the Lauren classification (Table 3). A clinical model was constructed based on these independent predictors. The ROC curve of this model showed AUC values of 0.70 (95% CI, 0.62–0.76), 0.67 (95% CI, 0.55–0.77), 0.65 (95% CI, 0.52–0.78), and 0.73 (95% CI, 0.59–0.86) in the training set, internal validation set, external test set-1, and external test set-2, respectively (Fig. 4).

**Table 3:** Results of univariate and multivariate logistic regression analysis

Characteristics	Univariable analysis		Multivariable analysis	
	OR	*p*-value	OR	*p*-value
Age (years)	0.96 (0.94, 0.99)	0.017	0.95 (0.92, 0.98)	0.004
Gender (female vs. male)	0.68 (0.37, 1.26)	0.225	NA	NA
Smoking history (absent vs. present)	1.08 (0.58, 2.01)	0.800	NA	NA
Drinking history (absent vs. present)	0.95 (0.50, 1.82)	0.879	NA	NA
Hypertension (yes vs. no)	0.79 (0.45, 1.39)	0.407	NA	NA
Diabetes (yes vs. no)	0.80 (0.35, 1.80)	0.584	NA	NA
CEA (ng/mL)	1.00 (0.99, 1.01)	0.875	NA	NA
CA125 (U/mL)	1.05 (1.01, 1.09)	0.011	1.05 (1.01, 1.10)	0.018
CA19-9 (U/mL)	1.00 (1.00,1.00)	0.369	NA	NA
Maximum tumor diameter (cm)	1.26 (1.12,1.43)	< 0.001	1.25 (1.10, 1.43)	< 0.001

Data in parentheses are 95% CI

*CEA* carcinoma embryonic antigen, *CA125* cancer antigen 125, *CA19-9* cancer antigen 19-9, *NA* not applicable, *OR* odds ratio

### Results of radiomics feature selection and model evaluation

**Fig. 5:**
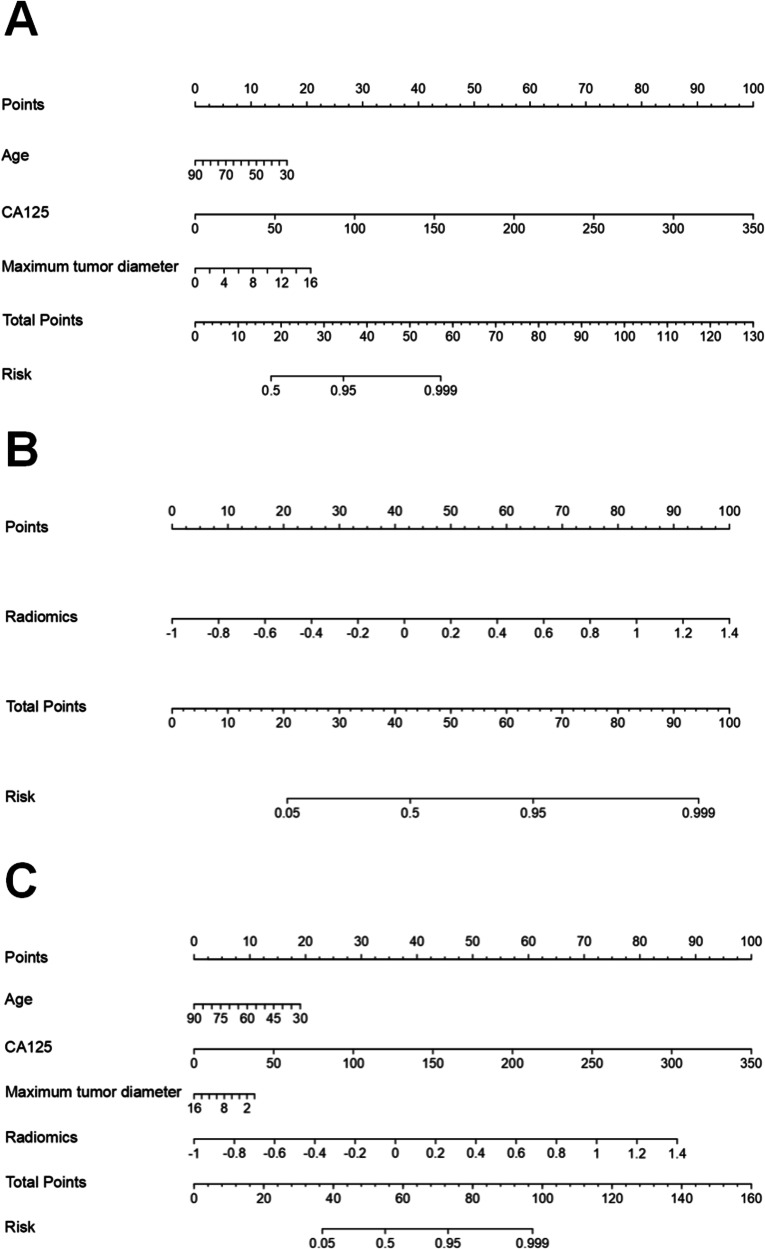
Nomogram developed from the training set for preoperative prediction of -GC the Lauren classification: **A** Clinical model, **B** Radiomics model, and **C** Combined model

During feature selection, Pearson correlation analysis initially revealed 347 features. The SelectKBest function further reduced this number to 134 features. Finally, the LASSO regression algorithm selected 7 optimal features (Supplementary Fig. 2). These features were used to calculate the Radscore, and a radiomic model was constructed using the LR classifier. The ROC curve for this model showed AUC values of 0.86 (95% CI, 0.81–0.91), 0.84 (95% CI, 0.75–0.93), 0.83 (95% CI, 0.71–0.93), and 0.81 (95% CI, 0.68–0.93) in the training set, internal validation set, external test set-1, and external test set-2, respectively (Fig. 4). A visual nomogram was developed on the basis of the training set (Fig. 5). The calibration curves and decision curves are shown in Supplementary Figs. 3 and 4.

### Evaluation of the combined clinical-radiomics model

A combined clinical‒radiomics model was constructed based on age, CA125 level, maximum tumor diameter, and the radiomics Radscore. The ROC curve for this model showed AUC values of 0.87 (95% CI, 0.82–0.92), 0.81 (95% CI, 0.71–0.89), 0.81 (95% CI, 0.7–0.91), and 0.82 (95% CI, 0.69–0.93) in the training set, internal validation set, external test set-1, and external test set-2, respectively (Fig. 4).

### Comparison of the performance of clinical, radiomics, and combined models

The AUCs of the clinical, radiomic, and combined models were compared using the DeLong test. The results revealed that in the training set, internal validation set, and external test set-1, the AUCs of the radiomic and combined models were significantly greater than those of the clinical model (*p* < 0.05). However, there was no significant difference in AUC performance between the radiomic and combined models (*p* > 0.05). In the external test set-2, there was no significant difference in AUC performance among the clinical, radiomics, and combined models (*p* > 0.05) (Supplementary Table 1). In summary, the radiomic and combined models generally outperformed the clinical model in terms of AUC performance, but there was no significant difference between the radiomic and combined models.

## Discussion

Preoperative prediction of the Lauren classification in patients with GC helps improve the specificity and effectiveness of treatment. The main findings of our study included the following: (1) efficient automatic segmentation of GC lesions could be achieved using the nnU-Net model; (2) preoperative identification of the Lauren classification in GC using a radiomic model could be achieved with high accuracy; and (3) the radiomic model for Lauren classification in GC had strong generalizability in a multicenter study.

The Lauren classification is an important prognostic factor for GC [26–28]. The preoperative classification relies primarily on histopathological results from an endoscopic biopsy. However, the limited sample size and uncertainty in sampling locations lead to a consistency rate of only 64.7–75% between biopsy and surgical samples, increasing the risk of misclassification [29, 30]. Studies indicate that intestinal-type GC generally has a better response to surgery and chemotherapy, whereas diffuse-type GC is more aggressive and has a poorer prognosis [3, 31]. Therefore, accurately predicting the Lauren classification preoperatively is crucial for clinicians to formulate individualized treatment plans and assess patient prognosis. In this study, we used a combination of nnU-Net and radiomics for noninvasive preoperative classification, with a focus on predicting the intestinal and diffuse types of GC. Given the similar survival outcomes between mixed-type and diffuse-type GC (45.6% vs. 43.4%) [27] and the complex biological characteristics of mixed-type GC, we chose to exclude it to enhance the model’s predictive performance.

To improve the efficiency and quality of radiomics research, this study developed an automatic segmentation model for GC lesions based on nnU-Net, achieving mean DSCs of 0.81 and 0.79 in the validation and test sets, respectively, indicating high efficiency and accuracy in segmenting GC tumor regions. Ferrante et al [32] applied a nnU-Net-based framework for automatic lung tumor segmentation on CT images, achieving a mean DSC of 0.78 ± 0.12. Jeon et al [33] developed a fully automated multiorgan segmentation tool for both plain and enhanced abdominal CT scans using a deep learning algorithm based on nnU-Net, with mean DSCs exceeding 0.94 for the liver, spleen, right kidney and left kidney. In contrast, Gao et al [34] developed a nnU-Net-based automatic segmentation model to segment lesions in patients with Crohn’s disease (CD), achieving a mean DSC of 0.824 in the test set. Additional studies [17, 35–37] have confirmed the high performance of automatic segmentation models. Collectively, these studies demonstrate that nnU-Net performs well in the automatic segmentation of both tumorous and inflammatory lesions, providing strong support for the present study.

Radiomics was first proposed by Lambin et al [9] and later applied by Aerts et al [10] to the analysis of CT imaging data for predicting tumor gene expression patterns and patient outcomes. In this study, we developed a radiomics model based on nnU-Net automatic segmentation to predict the Lauren classification of GC preoperatively. The results demonstrated that automatic segmentation has significant advantages in achieving efficient and accurate 3D-ROI segmentation of GC lesions [38, 39]. The AUC values of our clinical model, radiomics model, and combined model ranged from 0.65 to 0.73, 0.81 to 0.86, and 0.81 to 0.87, respectively, across different datasets. Delong tests indicated that the AUC performance of the radiomic and combined models was superior to that of the clinical model, with no significant difference between the radiomic and combined models. These findings suggest that radiomic features may already contain primary predictive information and that the addition of clinical features did not significantly enhance the performance of the combined model. Previous studies [40, 41] have also reported the strong performance of radiomic models in predicting the Lauren classification of GC, which is consistent with the findings of our study. For example, Nie et al [40] reported AUC values of 0.832 and 0.760 for venous-phase radiomic models in the training and test cohorts, respectively, with their combined model achieving AUCs of 0.849 and 0.793, respectively. Similarly, Li et al [41] reported AUCs of 0.855 and 0.802 for their combined radiomics model in the training and testing sets, respectively.

Unlike previous single-center studies [40–42], our study adopted a multicenter research design, providing stronger generalizability and broader applicability of the results. Multicenter studies can offer more diverse data sources, reducing sample bias and enhancing the reliability and external validity of the research results. However, multicenter studies also face challenges in coordinating and standardizing data collection and processing, requiring rigorous study design and management to ensure data consistency and comparability.

This study has several limitations. First, as a retrospective study, data collection and analysis may have biases. Second, although we introduced multicenter data for external testing, the sample size was relatively small. Future studies with larger-scale prospective research are needed to further validate the results. Third, our study only used CT imaging data; future research could combine other imaging modalities (such as MRI and PET) to further optimize and validate the model. Additionally, integrating more molecular characteristics, such as gene expression and proteomics data, could lead to the construction of multilayered, multidisciplinary comprehensive prediction models.

In conclusion, our study successfully developed a preoperative Lauren classification prediction model for GC based on nnU-Net automatic segmentation and radiomics technology. Through multicenter data validation, the model demonstrated high predictive performance, aiding clinicians in the early identification of GC classification and formulation of appropriate treatment plans to achieve precision and personalized medicine.

## Supplementary information

ELECTRONIC SUPPLEMENTARY MATERIAL

## Data Availability

The data supporting the findings of this study are available from the corresponding author upon reasonable request. Due to privacy or ethical restrictions, the data cannot be publicly shared.

## Abbreviations

AUC: Area under the curve
CA125: Cancer antigen 125
CA19-9: Carbohydrate antigen 19-9
CEA: Carcinoembryonic antigen
CT: Computed tomography
DSC: Dice similarity coefficient
GC: Gastric cancer
IoU: Intersection over union
LASSO: The least absolute shrinkage and selection operator
Radscore: Radiomics score
ROC: Receiver operating characteristic
ROI: Region of interest
