# Supplementary material for: Preoperative prediction of the Lauren classification in gastric cancer using automated nnU-Net and radiomics: a multicenter study
Source: Insights Imaging. 2025 Feb 25;16:48. doi: 10.1186/s13244-025-01923-9 (PMC11861772; doi:10.1186/s13244-025-01923-9)
Supplement: Supplementary file 1 — ELECTRONIC SUPPLEMENTARY MATERIAL [file 13244_2025_1923_MOESM1_ESM.pdf]

Preoperative Prediction of the Lauren Classification in Gastric Cancer Using Automated nnU-Net and Radiomics: A Multicenter Study

ELECTRONIC SUPPLEMENTARY MATERIAL

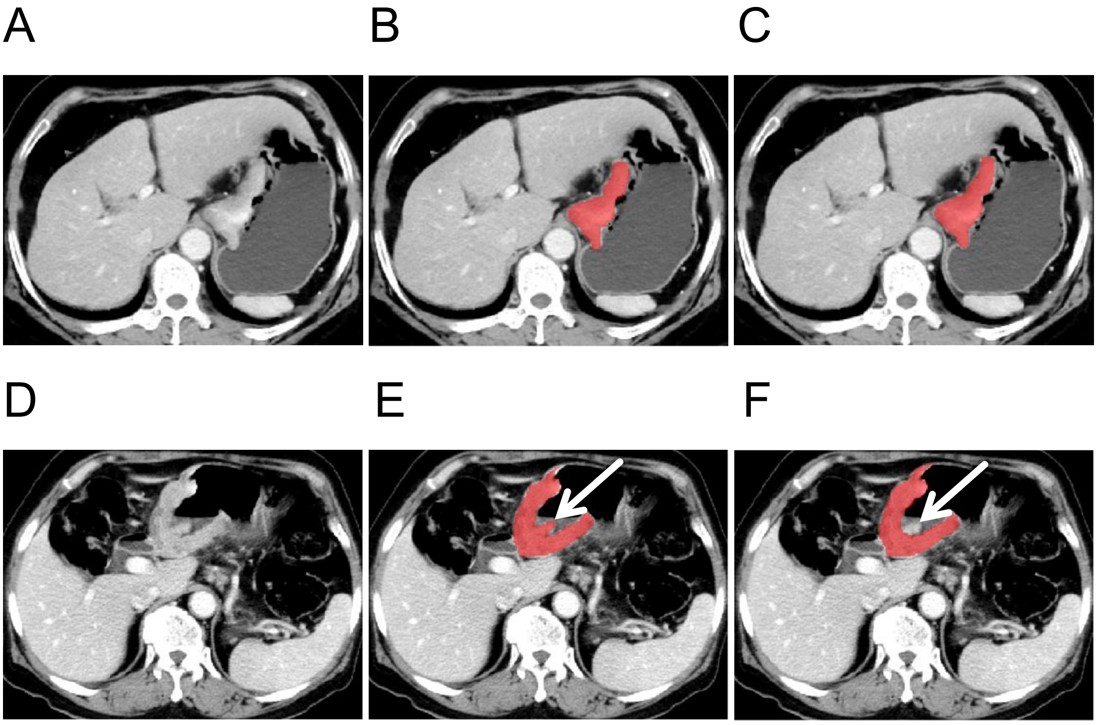

Supplementary Figure 1 Practical Application of the nnU-Net Automatic Segmentation Model in GC: Comparison between Manual Annotation and Automatic Segmentation. Figures (A-C) show the same patient, female, 76 years old, with a Dice Similarity Coefficient of 0.91. Figures (D-F) show another patient, female, 72 years old, with a Dice Similarity Coefficient of 0.78. Figures (A) and (D) are original portal venous phase CT images; Figures (B) and (E) are manually annotated by radiologists; Figures (C) and (F) are annotated by the nnU-Net automatic segmentation model. Figure (C) shows the automatic segmentation closely matches the manual annotation in Figure (B), while Figure (F) shows a tumor area not annotated by the automatic model (indicated by a white arrow).

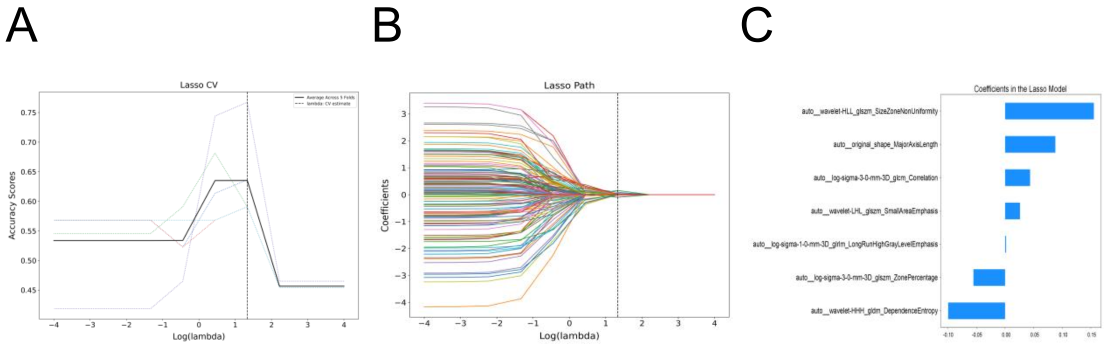

Supplementary Figure 2. Feature selection through Lasso regression analysis. (A) Cross-validation path for determining the optimal regularization parameter lambda. (B) Path of Lasso regression coefficients. (C) Feature Coefficients in the Lasso model.

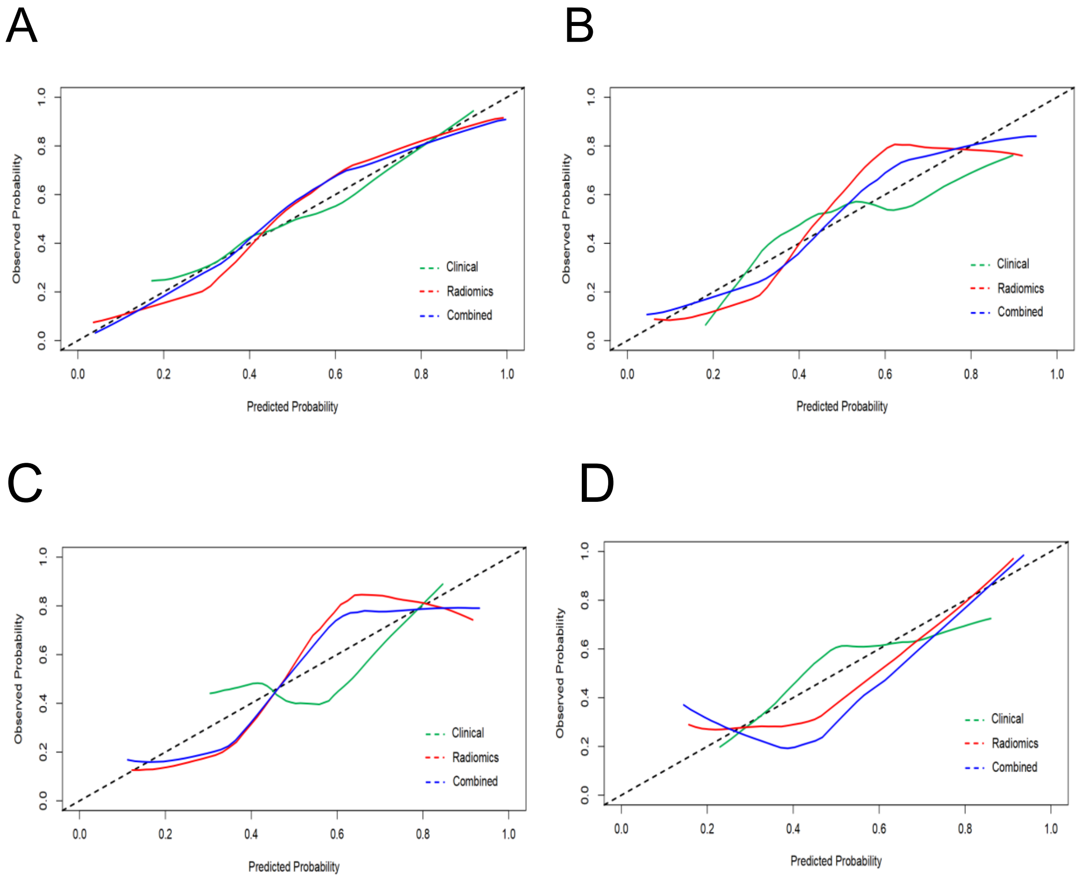

Supplementary Figure 3 Comparison of calibration plots for different models. (A) Training set; (B) Internal validation set; (C) External test set 1; (D) External test set 2. The dashed line represents perfect calibration. The solid lines represent the observed versus predicted probabilities. The green, red, and blue lines represent the clinical model, radiomics model, and combined model, respectively.

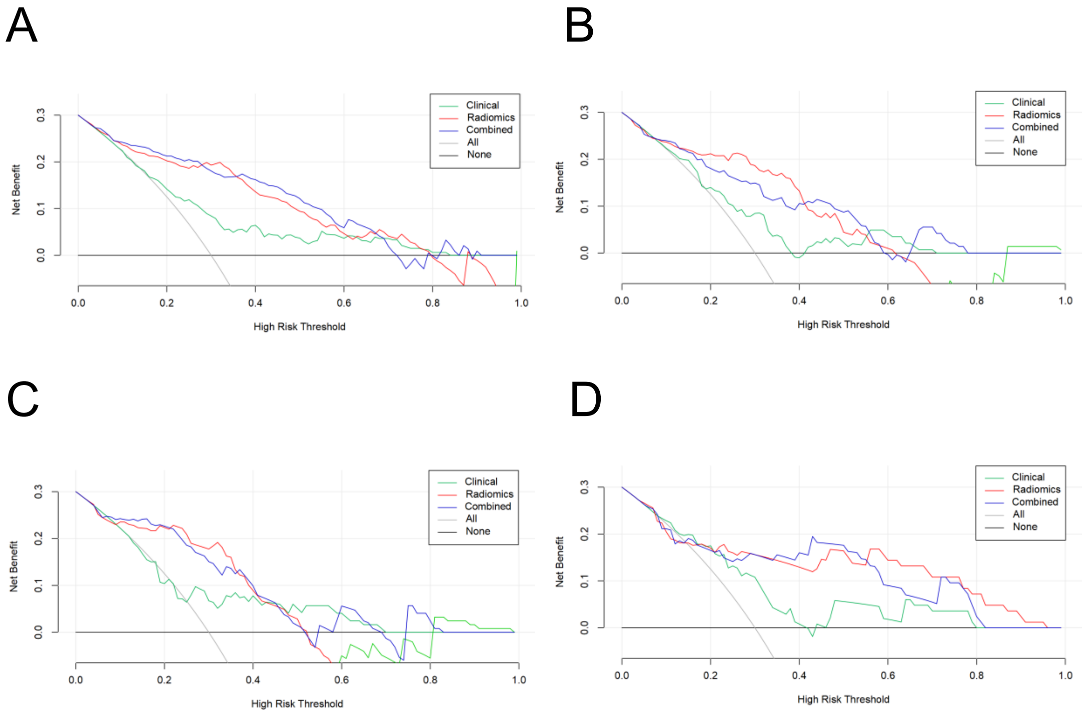

Supplementary Figure 4 Comparison of decision curves for different models. (A) Training set; (B) Internal validation set; (C) External test set 1; (D) External test set 2. The green, red, and blue lines represent the clinical model, radiomics model, and combined model, respectively.

Supplementary Table 1. DeLong Test Results Between Different Datasets and Models

Datasets	Comparison Models	*P* value
Training Set	Radiomics vs Clinical	＜0.001
	Combined vs Clinical	＜0.001
	Radiomics vs Combined	0.731
Internal Validation Set	Radiomics vs Clinical	0.004
	Combined vs Clinical	0.025
	Radiomics vs Combined	0.548
External Test Set 1	Radiomics vs Clinical	0.018
	Combined vs Clinical	0.029
	Radiomics vs Combined	0.857
External Test Set 2	Radiomics vs Clinical	0.331
	Combined vs Clinical	0.292
	Radiomics vs Combined	0.935
